# Operations for Necrosis

**Published:** 1855-05

**Authors:** J. C. Hughes

**Affiliations:** Professor of Surgery in the Iowa Medical Department


### NECROSIS—TWO CASES SUCCESSFULLY OPERATED UPON.

BY J. C. HUGHES, M. D.,

Professor of Surgery in the Iowa Medical Department.

CASE I.—TRAUMATIC NECROSIS OF THE TIBIA.—Mr. Eagleson, aged 21, a barber in this city, had been for the last seven years suffering from this disease.

It was the result of injury, the patient having received as he supposed, a slight wound in the integument, over the middle third of the tibia, which was followed by ostitis, and finally resulted in general necrosis of the anterior half of the middle, and lower portion of the upper third of the bone. The patient (as in most cases of the kind) did not allow it to interfere with his business, and continued to impose upon the limb from year to year, applying during this time all the healing nostrums of the day, but without any beneficial result, when from the increased suffering, as the disease more rapidly advanced, he was compelled to adopt a different course, and consulted me upon the subject.

I found upon examination, the leg to be almost twice its natural size at the diseased point. The integument, particularly over the affected part, was dense and hard, as well as much thickened and enlarged, a condition caused by the infiltration of plastic exudation, the result no doubt of frequent inflammatory attacks. Continuing my examinations with a probe, I found several cloacæ situated in the new bony deposit which formed a kind of shell, or bark, over the diseased portion of the tibia. Through these I introduced the probe, and could pass it to the depth of more than an inch, where I could distinctly feel the rough projections as well as detached and loose sequestra, which occupied the anterior of the bone. The only chance for saving the limb was by an operation, which I advised, and to which the patient reluctantly consented.

Dec. 1st, he was brought before the Medical Class for operation, and after the administration of chloroform, given at his own request, I commenced by making an incision with a scalpel to the extent of five inches along the course of the spine of the tibia. After separating the integument, which was of almost a bony hardness, I made a further examination through the cloaca and found it necessary to remove a portion of the external bone, as the natural openings in it were not sufficiently large to permit the passage of the sequestra which were situated within. I proceeded with chisels and mallet, which though it might seem a barbarous mode of surgical operation, was in this case absolutely demanded, to make a sufficient opening through which I was enabled to remove the greatest portion of the dead bone; but from the depth and position of some of the sequestra, I found it necessary to resort to the use of the trephine, before being able to remove all the fragments.

Several pieces of dead bone were removed, the largest measuring over two inches in length. The water dressings, accompanied by mild constitutional remedies, constituted the after treatment, and in two weeks the patient was able to leave the city, and in four weeks after the operation, was entirely well, the leg having resumed its normal size and natural healthy color.

This disease may be idiopathic or compound; or, as in the present case, traumatic, having been caused by a blow resulting it may have been in simple abscess, then periostitis and ostitis. It may make its appearance externally or internally, the former most common as arising from external causes, the latter from the result of constitutional difficulties, as from scrofulous, mercurial or syphilitic taints of the system.

It may occur at any period of life, but more frequently in the young, those under twenty years. The bones which are most liable to suffer are those which are most exposed, as the tibia, radius, and ulna, inferior maxillary and clavicle. All portions of the same bone are not alike susceptible to the disease, the more dense part generally suffering, while in the spongy portion it is very rare, caries being of more frequent occurrence in the cancellated texture.

Necrosis has not been thoroughly understood until within the last quarter of a century, by the majority of the profession, and even at this day of medical progress, you will find many who confer upon it, particularly if located near the joints, the name of white swelling. Where it is dependent upon a constitutional difficulty, the short bones are more liable to become affected, and I have seen cases where almost every bone had suffered. In other conditions we may have the surface or outer shell alone affected; or the inner portion adjacent to the medullary membrane becomes the seat of disease, or it may be general, affecting the entire bone. When the general constitutional cachexy is present, efforts of nature at repair, if they occur at all, are very feeble, and in nine cases out of ten, removal of the limb is demanded.

CASE II.—NECROSIS—AMPUTATION.—Mr. Reeves, aged about 40, a prominent citizen of Sigourney, Keokuk county, of this State, had for 25 years been suffering from the same disease. It first attacked the tibia, having presented the ordinary symptoms of inflammation attendant upon ostitis; exfoliation from the external surface followed, finally the deeper parts became affected, and thus the disease continued from year to year, until the constitutional symptoms presented themselves accompanied by hectic fever. Finding himself sinking under the disease, he applied to me in January last for advice. Upon examination, I found the disease to have extended to the lower extremity of both the tibia and fibula, also the tarsal and metatarsal bones, and at once advised an operation, to which he willingly consented, having previously made up his mind that the leg must and should come off.

On January 4th, he presented himself before the Class, and after administering chloroform, I proceeded by the usual mode of the flap operation to remove the leg at about two and a half inches below the knee. For a few days after the operation his symptoms were not the most favorable. But the primary cause being now removed, he soon began to improve, and in some four weeks was able to return home, and from letters which I have since received from him, he speaks of his general good health, and rejoices that he has been relieved of so much suffering.

While this disease affects the external surface alone, exfoliation may take place, followed by restoration of the parts to a healthy condition, or if internal, the result though more tedious, may be the same; or even should it be general, the whole thickness of the bone being affected, there is a possibility of natures accomplishing a cure. But in all these cases, it is the duty of the surgeon to watch carefully the case during the progress of the local death, and when dead, and nature willing and anxious to throw it off, to interfere and remove it as you would any other foreign substance from the body.

